# Factors associated with the use of benzodiazepine and opioid prescription drug in the student population: a cross-sectional study

**DOI:** 10.1038/s41598-024-63037-4

**Published:** 2024-06-06

**Authors:** Charlotte Thomas, Thibaut Dondaine, Clément Caron, Axel Bastien, Nathalie Chérot, Sylvie Deheul, Sophie Gautier, Olivier Cottencin, Sophie Moreau-Crépeaux, Régis Bordet, Louise Carton

**Affiliations:** 1 Department of Psychiatry and Addiction Medicine, CHU Lille, Lille, France; 2 University of Lille, Inserm, CHU Lille, Lille Neuroscience and Cognition, Degenerative and Vascular Cognitive Disorders, UMR-S1172, Lille, France; 3 IMPECS - IMPact de l’Environnement Chimique sur la Santé humaine, Department of Occupational Health, University of Lille, CHU Lille, Institut Pasteur de Lille, ULR 4483, 59000 Lille, France; 4 CHU Lille, Pharmacology Department, Pharmacovigilance and Addictovigilance center, Lille, France; 5 Lille Neuroscience & Cognition Centre (LiNC), Department of Psychiatry and Addiction Medicine, University of Lille, CHU Lille, INSERM U-1172, Plasticity & SubjectivitY (PSY) team, Lille, France; 6 University of Lille, University student health services, Lille, France

**Keywords:** Health care, Medical research, Risk factors, Target identification

## Abstract

The misuse of benzodiazepines and opioid medications is frequent in students. To improve our understanding of this behavior, we aimed to identify factors associated with separate and concomitant use of these substances. Anonymous self-reported questionnaires were e-mailed to students enrolled at a French university between March and July 2021, covering: sociodemographic characteristics, academics, psychoactive substance use, ADHD symptomatology (adulthood and childhood), and psychiatric/psychological or addiction follow-up. Factors associated with the use of benzodiazepines and opioid medications included female sex (OR = 1.41 [1.08; 1.86]) and OR = 1.38 [1.06; 1.79], respectively), older age (OR = 1.65 [1.04; 2.6] and OR = 2.17 [1.4; 3.36], respectively), current psychiatric/psychological follow-up (OR = 6.53 [5.18; 8.24] and OR= 1.5 [1.12; 2.0], respectively), ADHD symptomatology (OR= 2.33 [1.71;3.16] and OR= 1.61 [1.15; 2.24], respectively), polyconsumption (tobacco use for benzodiazepine users, OR = 1.38 [1.04; 1.82]; alcohol use OR = 1.67 [1.17; 2.39] and tobacco use OR = 1.62 [1.23; 2.14] for opioid users). These factors were even more strongly associated with the concomitant use of benzodiazepines and opioid medications: older age (OR = 3.64 [2.22; 5.99]), female sex (OR = 1.54 [1.1; 2.14]), grade repetition (OR = 1.7 [1.14; 2.54]), psychiatric/psychological follow-up (OR = 4.51 [3.35;6.06]), ADHD symptomatology (OR = 5.3 [3.69; 7.63]), polyconsumption (tobacco use OR = 2.05 [1.39; 3] and cannabis use, OR = 2.07 [1.97; 4.16]. The factors associated with the use of benzodiazepines and prescription opioids identified in this study could lead to the development of targeted prevention methods.

## Introduction

In recent years, the prevalence of the use of benzodiazepines and opioid medications has increased in the general population, leading to a significant increase in the risk of dependence and death from overdose^1–4^. Benzodiazepines are part of the symptomatic treatments for anxiety and sleep disorders^5^. While they are frequently prescribed around the world, they are associated with several adverse effects, such as sedation, cognitive disorders, rebound anxiety upon discontinuation, paradoxical reactions, and a high risk of drug dependence^5,6^. Opioid medications are mainly used for their analgesic properties but can also be used for recreational purposes^5^. There are many undesirable effects associated with opioid medication use, including substance use disorders, sedative effects, respiratory depression, and overdose^7^.

The combined use of benzodiazepines and opioid medications is particularly concerning among students. The psychic vulnerability of this population, as well as the stress of studying, parties in search of sensations and new experiences, and even cognitive or physical doping, are all reasons for the emergence of new risk behaviors in relation to substances^8–13^. A paradigm shift has been observed over the last few decades, with new illicit substances, earlier onset of use, easy access for young people via the Internet, but also changes in consumption patterns^8,9,12,14,15^. In the case of prescribed substances in particular, there is an increase in misuse in young people for automedication, cognitive doping or recreational purposes, sometimes involving “doctor shopping” behavior^8,11,14,15^. The “Purple Drank” phenomenon reflects this last point. This codeine syrup-based drink, used recreationally in student circles, led to a legislative tightening of prescription opioids distribution in France in 2017^8,15–18^. A review published in 2021 highlighted the vulnerability of adolescents and young adults to over-the-counter medication misuse, reminding doctors and pharmacists of their role in identifying and educating patients^15^. Similarly, cognitive doping practices are frequent in student circles^8,11,19,20^, involving psychostimulants but also anxiety-reducing drugs^8,11,19^. Accordingly, students with academic difficulties would benefit from being identified by addictology prevention programs^19^. This is particularly true for student with attention deficit hyperactivity disorder (ADHD) condition, an often-under-diagnosed neurodevelopmental disorder that is frequently associated with substance use disorders and can have significant repercussions at school^19,21,22^.

From an epidemiological point of view, literature data corroborate the prevalent use of benzodiazepines and opioid drugs among students. In the USA, a literature review published in 2022 found that the prevalence of opioid medications misuse among students ranged from 4 to 19.7%^23^. The students with the highest level of risk were those who reported high rates of psychological distress, depression, and suicidal ideation or behavior as well as consumption of alcohol and illicit substances^23^. Additionally, a cross-sectional analysis conducted between 2015 and 2016 in the USA found a higher rate of benzodiazepine misuse among 18- to 25-year-old individuals than among other age group. Opioid medications were the substances most associated with the consumption of benzodiazepines^24^. In 2021, a study carried out in the USA examined a sample of students and found a significant association between benzodiazepine consumption and nonmedical prescription opioid use^25^. In 2017, a cross-sectional study carried out on 46 203 French students aimed to assess the use of psychoactive substances among 18- to 25-year-old individuals. Prescription substances ranked third among women and fourth among men. Among prescribed substances, opioids were in first place (15%), followed by benzodiazepines (3.4%)^8^.

In line with these results, we performed a previous pharmacoepidemiological study, named PETRA, that found that the most common prescription drugs used by students since their first year of study were benzodiazepines and opioid medications^26^. In addition, one-third of benzodiazepine users and approximately 20% of opioid users in this study reported that they had already felt a sense of dependence on the substance^26^. However, in this article, the data were essentially descriptive and did not provide any additional information on possible factors associated with such consumption. Given the high prevalence of benzodiazepines and opioid drugs use in our student sample, we felt it was important to characterize this use and the factors associated with it, to better target prevention programs. In addition, we wondered whether the concomitant use of these substances might be associated with particular risk factors.

Therefore, our objective was to identify factors associated with benzodiazepine and opioid drug use separately and concomitantly in the student population based on our previous study PETRA sample. More specifically, we also wanted to assess the link with ADHD symptoms.

## Material and methods

### Study design

This work is an ancillary study of the PETRA study, an observational, cross-sectional survey conducted between March and July 2021 at the University of Lille, France.

### Population

The inclusion criteria were: enrollment at the University of Lille during the 2020–2021 academic year regardless of the university course, and voluntary participation. The exclusion criterion was age under 18 years at the time of the study.

### Measures

A self-administered questionnaire was e-mailed to all students, with three subsequent reminders. Students were asked to provide data regarding their sociodemographic characteristics, academics, consumption of benzodiazepines and opioid medications, tobacco use, alcohol use, cannabis use, and current presence of associated psychiatric/psychological or addiction follow-up. Substance use was defined as having used the substance at least once since the first year of university. We have chosen to explore tobacco, cannabis and alcohol as associated substances, as they are known in the literature to be the most frequently experienced substances among young people^11,21,27^. We also assessed whether opioid medications and benzodiazepines use could be linked to ADHD. To this end, we administered the Adult ADHD Self-Report Scale (ASRS-6) and Wender Utah Rating Scale (WURS-25), which are self-reported scales that are used to screen for ADHD among adults and children, respectively^28,29^. The ASRS-6 is considered positive when the score is greater than or equal to 4, and the WURS-25 is considered positive when the score is greater than or equal to 46. When both scales were negative (ASRS- WURS-), we considered that there were no ADHD symptoms. If symptoms were present only in adulthood (ASRS+ WURS-), we considered that substance use could induce psychomotor symptoms. If symptoms were only present during childhood (ASRS- WURS+), we considered that ADHD symptoms were in remission. Finally, if both scales were positive (ASRS+ WURS+), we considered that ADHD was persistent.

### Statistical analyses

We analyzed the use of benzodiazepine and/or opioid medication among a student population, considering numerous variables. We identified four groups grouped into a group variable: non users, benzodiazepine only users, opioid only users and combined users. Initially, we selected variables associated with group for inclusion in a multinomial model using chi-square tests. We then fitted a multinomial model using the `multinom()` function from the `nnet` package in R^30^, with the dependent variable `Group` predicted by several independent variables such as `Age`, `Sex`, `Family situation`, `Income`, `Housing`, `Current year`, `Repeated years`, `Advanced years`, `use of alcohol`, `use of cannabis`, `use of tabaco`, `ADHD symptomatology`, `Psychiatric/psychologic follow up`. In order to retain the most significant variables in the regression model, we used the backward stepwise method. To select the best model, we used the `stepAIC()` function from the `MASS` package in R^30^, which performs automated model selection based on the Akaike information criterion (AIC). The final model was then validated by evaluating its goodness of fit and predictive ability using appropriate measures. The results of the final model were presented using odds ratios with 95% confidence intervals. Overall, our statistical approach allowed us to determine the most relevant variables associated with the consumption patterns and provide valuable insights into the behavioral trends among the student population.

### Ethics

The PETRA study is a type 3 observational epidemiological study. Each student received information on the type of study and its objectives before completing the questionnaire. The need for informed consent was deemed unnecessary according to national regulations. Non-response to the questionnaire was considered opposition. The anonymity of the students was warranted both in the collection of data and in their analysis, respecting the MR003 standard of the Commission Nationale de l‘Informatique et des Libertés (CNIL). All methods were carried out in accordance with relevant guidelines and regulations. Students were given contact details for psychiatric and addiction services in case they felt they might have difficulty filling in the questionnaires (feeling of ill-being and/or anxiety, judgment of excessive consumption of psychoactive substances or disabling attentional symptomatology).

### Human ethics and consent to participate declarations

Ethical approval for the study was obtained from the Sud-Ouest Outre-Mer ethics committee (IORG0009855) under the number CPP2019_11_089/2019_A00316_51/19.10.04.61745.

## Results

### Sociodemographic characteristics of benzodiazepine and opioid prescription drug users

Nearly 80% of benzodiazepines, opioids drugs and combined users were female. For the most part, users were single (61.2% for benzodiazepines users, 60.1% for opioid drugs users and 52.5% for combined users) and aged between 21 and 25 (46.2%, 49% and 52.5% respectively). The majority received grants (41.9% for benzodiazepines users, 37.8% for opioids drugs, 35.5% for combined users) and had their own accommodation (53.1%, 52.1% and 61.5% respectively) (Table 1).

**Table 1:** Sociodemographic data of benzodiazepine users (BZD+OPI–), opioid drug users (BZD–OPI+), and combined users (BZD+OPI+) versus nonusers (BZD–OPI–) among students*.

	BZD–OPI–	BZD + OPI–	BZD–OPI+	BZD + OPI+	p-value
	N=3362 (77.6%)	N=418 (9.4%)	N=386 (8.7%)	N=265 (6%)	
Age (%)					<10ˉ^3^
18–20	1922 (57.2)	185 (44.3)	155 (40.2)	74 (27.9)	
21–25	1243 (37)	193 (46.2)	189 (49)	139 (52.5)	
>25	197 (5.9)	40 (9.6)	42 (10.9)	52 (19.6)	
Sex (%)					0.004
Female	2642 (78.6)	336 (80.4)	298 (77.2)	206 (77.7)	
Male	900 (26.8)	82 (19.6)	88 (22.8)	59 (22.3)	
Housing (%)					<10ˉ^3^**
Personal housing	1565 (46.5)	222 (53.1)	201 (52.1)	163 (61.5)	
Parents/guardian’s house	1493 (44.4)	158 (37.8)	155 (40.2)	75 (28.3)	
University residence	269 (8)	32 (7.7)	27 (7)	24 (9.1)	
Other	35 (1)	6 (1.4)	3 (0.8)	3 (1.1)	
Family situation (%)					<10ˉ^3^
Couple	1145 (34.1)	162 (38.8)	154 (39.9)	126 (47.5)	
Single	2217 (65.9)	256 (61.2)	232 (60.1)	139 (52.5)	
Income (%) (*multiple choices*)					<10ˉ^3^
Student job	151 (4.5)	19 (4.5)	32 (8.3)	23 (8.7)	
Grants	1266 (37.7)	175 (41.9)	146 (37.8)	94 (35.5)	
Paid internship	189 (5.6)	35 (8.4)	22 (5.7)	34 (12.8)	
Parental assistance	855 (25.4)	86 (20.6)	70 (18.1)	35 (13.2)	
No income	402 (12)	38 (9.1)	35 (9.1)	22 (8.3)	
Multiple	499 (14.8)	81 (19.4)	81 (21)	57 (21.5)	

*BZD* benzodiazepines, *OPI* opioid medications.

*Participants are considered users if they have used the substance at least once since their first year of university.

**p-value calculated using the Fisher test.

### Academic characteristics of benzodiazepine and opioid prescription drug users

Most students using benzodiazepines, opioid drugs, and them both were in their first year of study (34.9%, 28.8% and 24.6% respectively), in Art-Literature and Language and Human and Social Sciences (ALL-HSS) (49.3%, 38.9% and 48.3% respectively) and preparing for a bachelor's degree (55.5%, 52.1% and 45.3% respectively) (Table 2).

**Table 2:** Academic data of benzodiazepine users (BZD+OPI–), opioid drug users (BZD-OPI+), and combined users (BZD+OPI+) versus nonusers (BZD–OPI–) among students*.

	BZD–OPI–	BZD + OPI–	BZD–OPI+	BZD + OPI+	p-value
	N=3362 (77.6%)	N=418 (9.4%)	N=386 (8.7%)	N=265 (6%)	
Field of study (%)					<10ˉ^3^
Aar literature and language, human and social sciences	1216 (36.2)	206 (49.3)	150 (38.9)	128 (48.3)	
Law economics and management	551 (16.4)	58 (13.9)	72 (18.7)	30 (11.3)	
Health	942 (28)	99 (23.7)	94 (24.3)	66 (24.9)	
Sports	54 (1.6)	1 (0.2)	3 (0.7)	2 (0.7)	
Technological sciences and IUT	599 (17.8)	54 (12.9)	67 (17.4)	39 (14.7)	
Degree (%)					<10ˉ^3^**
Bachelor	1788 (53.2)	232 (55.5)	201 (52.1)	120 (45.3)	
Master’s	620 (18.4)	81 (19.4)	93 (24.1)	78 (29.4)	
Health studies’ first year	269 (8)	15 (3.6)	15 (3.9)	7 (2.6)	
Medicine/pharmacy/maieutic/dentist	459 (13.7)	59 (14.1)	54 (14)	44 (16.6)	
Nurses. speech therapist. orthoptics	56 (1.7)	11 (2.6)	12 (3.1)	3 (1.1)	
Doctorate	42 (1.2)	6 (1.4)	7 (1.8)	7 (2.6)	
Other	128 (3.8)	14 (3.3)	4 (1)	6 (2.3)	
Current year (%)					<10ˉ^3^
1st year	1383 (41.1)	146 (34.9)	111 (28.8)	63 (24.6)	
2nd year	713 (21.2)	88 (21.1)	83 (21.5)	50 (18.9)	
3rd year	517 (15.4)	71 (17)	83 (21.5)	43 (16.2)	
4th year	308 (9.2)	41 (9.8)	51 (13.2)	36 (13.6)	
5th year	297 (8.8)	44 (10.5)	39 (10.1)	40 (15.1)	
6 year and plus	144 (4.3)	28 (6.7)	19 (4.9)	33 (12.5)	
Repeated years (%)					<10ˉ^3^
0	2341 (69.6)	249 (59.6)	225 (58.3)	130 (49.1)	
1	749 (22.3)	113 (27)	109 (28.2)	75 (28.3)	
2 or more	272 (8.1)	56 (13.4)	52 (13.5)	60 (22.6)	
Advanced years (%)					0.51**
0	2987 (88.8)	370 (88.5)	339 (87.8)	226 (85.3)	
1	327 (9.7)	44 (10.5)	43 (11.1)	33 (12.5)	
2 or more	48 (1.4)	4 (1)	33 (8.5)	6 (2.3)	

*BZD* benzodiazepines, *OPI* opioid medications, *IUT* University Technology Institute.

*Participants are considered users if they have used the substance at least once since their first year of university.

**p-value calculated using the Fisher test

### Medical characteristics and associated consumption of benzodiazepine and opioid prescription drug users

Half of users of benzodiazepines alone or in combination with opioid drugs were involved in psychiatric follow-up, compared with 20% of users of opioid drugs alone. The prevalence of addictive follow-up was around 3% for benzodiazepines and opioid use, and 6.4% for combined use. Concerning ADHD symptomatology, around one third of benzodiazepines, opioid drugs and concomitant users scored positive on the ASRS alone (ASRS+ WURS-), in favor of substance-related psychocomportemental symptoms. 22.2% of benzodiazepine users, 14.8% of opioid drugs users and 33.2% of combined users reported lifetime ADHD symptoms (ASRS+ WURS+), in contrast to 10% in non-users. A large majority of users had already consumed alcohol since starting university: 86.1% for benzodiazepine users, 89.1% for opioid drugs users and 91.3% for combination users. More than half of benzodiazepine and opioid users had already tried tobacco since the start of university, and nearly three-quarters in the case of combined use. 43.8% of benzodiazepine users, 46.9% of opioid drugs users and 69.1% of combined users had already tried cannabis (Table 3).

**Table 3:** Medical and associated consumption data of benzodiazepine users (BZD+OPI–), opioid drug users (BZD–OPI+), and combined users (BZD+OPI+) versus nonusers (BZD–OPI–) among students*.

	BZD–OPI–	BZD + OPI–	BZD–OPI+	BZD + OPI+	p-value
	N=3362 (77.6%)	N=418 (9.4%)	N=386 (8.7%)	N=265 (6%)	
Psychiatric follow-up (%)	401 (11.9)	212 (50.7)	75 (19.4)	124 (46.8)	<10ˉ^3^
Addictive follow-up (%)	71 (2.1)	13 (3.1)	10 (2.6)	17 (6.4)	<10ˉ^3^
ADHD symptomatology					
WURS–ASRS–	2070 (61.6)	163 (39)	195 (50.5)	80 (30.2)	<10ˉ^3^
WURS–ASRS+	815 (24.2)	138 (33)	107 (27.7)	77 (29.1)	
WURS+ ASRS–	135 (4)	24 (5.7)	27 (7)	20 (7.5)	
WURS+ ASRS+	342 (10.2)	93 (22.2)	57 (14.8)	88 (33.2)	
Related substances (%)					
Alcohol	2598 (77.3)	360 (86.1)	344 (89.1)	242 (91.3)	<10ˉ^3^
Tobacco	1165 (34.7)	214 (51.2)	216 (56)	193 (72.8)	<10ˉ^3^
Cannabis	960 (28.6)	183 (43.8)	181 (46.9)	183 (69.1)	<10ˉ^3^

*BZD* benzodiazepines, *OPI* opioid medications, *ADHD* attention deficit hyperactivity disorder, *WURS* wender utah rating scales, *ASRS* adult ADHD self-report scale.

^*^Participants are considered users if they have used the substance at least once since their first year of university.

### Factors associated with benzodiazepine use

The factors associated with benzodiazepine use were: older age (OR = 1.3 [1.01; 1.67] for the 21–25 years age group, OR=1.65 [1.04; 1.86] for the 25 and older age groups), female sex (OR = 1.41 [1.08; 1.86]), having paid internship (OR = 1.76 [1.03; 3.03]), ADHD symptoms in adulthood only (ASRS+ WURS-, OR = 1.91 [1.48; 2.47]), ADHD symptoms in childhood only (ASRS- WURS+, OR = 1.87 [1.14; 3.05]) and persistent ADHD (ASRS+WURS+, OR = 2.33 [1.71; 3.16]) (Table 4).

**Table 4:** Multivariate analysis of factors associated with the use of opioid medication and/or benzodiazepine in the student population after selection of variables with the backward stepwise method.

	BZD + OPI–	BZD–OPI+	BZD + OPI+
	AOR (CI 95%)	AOR (CI 95%)	AOR (CI 95%)
Age			
18–20	Ref	Ref	Ref
21–25	1.3 (1.01; 1.67)	1.5 (1.17; 1.94)	1.86 (1.33; 2.62)
>25	1.65 (1.04; 2.6)	2.17 (1.4; 3.36)	3.64 (2.22; 5.99)
Sex			
Male	Ref	Ref	Ref
Female	1.41 (1.08; 1.86)	1.38 (1.06; 1.79)	1.54 (1.1; 2.14)
Family situation			
Single	Ref	Ref	Ref
Couple	1.11 (0.88; 1.39)	1.1 (0.88; 1.38)	1.45 (1.1; 1.92)
Income			
Parental help	0.98 (0.64; 1.51)	0.87 (0.56; 1.34)	0.75 (0.42; 1.37)
Grants	1.38 (0.93; 2.04)	1.27 (0.85; 1.89)	1.34 (0.79; 2.27)
Student job	1.08 (0.58; 2)	1.65 (0.97; 2.81)	1.61 (0.81; 3.18)
Paid internship	1.76 (1.03; 3.03)	0.88 (0.49; 1.58)	2.29 (1.21; 4.33)
Multiples	1.12 (0.71; 1.77)	1.34 (0.87; 2.08)	1.5 (0.85; 2.65)
Other	Ref	Ref	Ref
Repeated years			
0	Ref	Ref	Ref
1	1.29 (0.99; 1.67)	1.31 (1.02; 1.69)	1.33 (0.96; 1.86)
2 et plus	1.33 (0.93; 1.92)	1.3 (0.91; 1.87)	1.7 (1.14; 2.54)
Psychiatric/psychological follow-up	6.53 (5.18; 8.24)	1.5 (1.12; 2)	4.51 (3.35; 6.06)
Addictive follow-up	0.51 (0.27;0.97)	0.93 (0.46; 1.89)	1.19 (0.63; 2.26)
ADHD symptomatology			
ASRS- WURS–	Ref	Ref	Ref
ASRS+ WURS–	1.91 (1.48; 2.47)	1.38 (1.07; 1.79)	2.48 (1.75; 3,5)
ASRS+ WURS–	1.87 (1.14; 3.05)	1.92 (1.22; 3.02)	3.14 (1.78; 5.56)
ASRS+ WURS+	2.33 (1.71; 3.16)	1.61 (1.15; 2.24)	5.3 (3.69; 7.63)
Related substances			
Alcohol	1.29 (0.93; 1.8)	1.67 (1.17;2.39)	1.03 (0.62; 1.72)
Tobacco	1.38 (1.04; 1.82)	1.62 (1.23; 2.14)	2.05 (1.39; 3)
Cannabis	1.31 (0.98; 1.75)	1.31 (0.99; 1.72)	2.07 (1.97; 4.16)

*BZD* benzodiazepines, *OPI* opioid medication, *ADHD* attention deficit hyperactivity disorder, *WURS* wender utah rating scales, *ASRS* adult ADHD self-report scale, *AOR* adjusted odds ratio, *CI* confidence interval.

The presence of psychiatric follow-up (OR = 6.53 [5.18; 8.24]) and addiction follow-up (OR = 0.51 [0.27; 0.97]) were oppositely associated with benzodiazepine use since the first university year. Tobacco use was also found to be associated with benzodiazepine use (OR = 1.38 [1.04; 1.82]) (Table 4).

### Factors associated with opioid medications use

The factors associated with opioid medications use were: older age (OR = 1.5 [1.17; 1.94] for 21–25 years age group; OR = 2.17 [1.4; 3.36] for the 25 and older age group), female sex (OR = 1.38 [1.06; 1.79]), having repeated a year (OR = 1.31 [1.02; 1.69]), ADHD symptoms in adulthood (ASRS+ WURS-, OR = 1.38 [1.07; 1.79]), ADHD symptoms in childhood (ASRS- WURS+, OR = 1.92 [1.22; 3.02]) and persistent ADHD (ASRS+WURS+, OR = 1.61 [1.15; 2.24]) (Table 4).

The presence of psychiatric follow-up (OR = 1.5 [1.12; 2]) was associated with opioid drug use since the start of the first university year but not addiction follow-up. Both alcohol use (OR = 1.67 [1.17; 2.39]) and tobacco use (OR = 1.62 [1.23; 2.14]) were associated with opioid medication use (Table 4).

### Factors associated with combined benzodiazepines and opioid medication use

Factors associated with concomitant benzodiazepine and opioid medication use were: older age (OR = 1.86 [1.33; 2,62] for 18-25 years and OR = 3.64 [2.22; 5.99] for over 25s), female sex (OR = 1.54 [1.1; 2.14]), being in a couple (OR = 1.54 [1.1; 2.14]), having paid internship (OR = 2.29 [1.29; 4.33]), repeating two years and more (OR = 1.7 [1.14; 2.54]), ADHD symptoms in adulthood (ASRS+ WURS-, OR = 2.48 [1.75; 3.5]), ADHD symptoms in childhood (ASRS- WURS+, OR = 3.14 [1.78; 5.56]) and persistent ADHD (ASRS+WURS+, OR = 5.3 [3.69; 7.63]) (Table 4).

The presence of psychiatric follow-up was associated with concomitant benzodiazepines and opioid medications use (OR = 4.51 [3.35; 6.06]). Both tobacco (OR = 2.05 [1.39; 3]) and cannabis use (OR = 2.87 [1.97; 4.16]) were associated with concomitant use of these two substances (Table 4).

## Discussion

Overall, we found that factors associated with benzodiazepines use and opioid medications use were female sex, older age, positive ADHD screening, psychiatric/psychologic follow-up and associated substance use. These factors were even more strongly associated with concomitant use of benzodiazepines and opioids. Furthermore, concomitant use was associated with having repeated at least 2 years of school.

Our results showed that most benzodiazepines and opioid medication users were older than nonusers. In line with our results, an American literature review performed in 2020 found that age was positively associated with an increase in the prevalence of prescription drug misuse, rising from adolescence and peaking in early adulthood^31^. This reinforces the importance of intervening as early as possible to avoid a slide toward chronic use and drug dependence. The association between female sex and the use of benzodiazepines and opioid medication was also reported in previous studies: women have been shown to have higher levels of consumption of both drugs separately and concomitantly^3,8,32^. It remains unclear whether this difference between sexes is due to the reason for consumption, which varies across genders. In the general population, Maust et al. found that women consumed more benzodiazepines than men but were less prone to misuse^24^. Also, Schepis et al. found that men aged between 18 and 49 had lower rates of benzodiazepines misuse than women^33^. However, there was no significant association between gender and benzodiazepine misuse in the youngest age group, between the ages of 12 and 17^33^. Weyandt et al. reported that opioid misuse was more likely to be recreational among male students, while young women were more likely to self-medicate^23^. With regard to illicit substances, a cross-sectional study carried out on a sample of Italian high school students between 2014 and 2016 found a predominance of lifetime use among male individuals, as well as more poly-drug use^34^. The COSYs study also described a predominance of illicit substance use among male students compared to females^8^. In the same study, prescribed substances were more prevalent among young women, particularly during exam periods^8^. The association between type of substance and reason for use according to gender may merit further investigation in the future. Our results showed an association between being in a couple and the concomitant use of benzodiazepines and opioid drugs. This raises the question of whether the fact of having a partner who is a substance user, and the greater availability of the substance through this partner, may be at the root of this association. In 2010, a study showed that among women in a relationship, the fact that the partner had prescribed drugs encouraged misuse, not because the partner was a user, but because of easier access to the drug^35^. Among men, neither partner use nor relationship factors were associated with nonmedical use of prescription drugs after considering the impact of individual-level risk factors^35^. To our knowledge, the question of marital status and drug misuse in the specific population of adolescents and young adults is still poorly documented in the literature and could be further explored.

In our sample, we found that around 10% of non-user students showed positive symptomatology for the ADHD screening tests, which is higher than the known prevalence of ADHD in general population. This prevalence was higher among students using benzodiazepines and opioid drugs and tripled in the case of combined use. In addition, there was an association between repetition and the use of opioid drugs, and the combined use of opioid drugs and benzodiazepines. This is in line with the findings of the literature. In 2009, DuPaul et al. found a prevalence of 2% to 8% of students reporting clinically significant levels of ADHD symptomatology^36^. Furthermore, at least a quarter of students experiencing difficulties in their studies were diagnosed with ADHD^36^. In this study, the diagnosis of ADHD was generally ascertained using individual and parent clinical interviews, and multimethod assessment of ADHD with scales such as the WURS, the College ADHD Response evaluation System, and the internal restlessness scale^36^. In 2018, Romo et al. found that the prevalence of ADHD in a sample of French students was 5.6% using ASRS-6 and WURS-25 scales^22^. Furthermore, 42.2% of students with ADHD had already repeated a year in their studies, compared with a quarter of students in the general population^22^. An American study published in 2018 found a lifetime diagnostic prevalence of ADHD (self-reported) in the student population of 11.3%^21^. 40% of ADHD participants in this study reported using psychotropic medication^21^. In this vein, Di Nicola et al. showed, using a self-report survey, that addictive behavior was more prevalent among young people with difficulties at school^34^.The fact that positive screenings for ASRS and WURS, and grade repetition were associated with benzodiazepines and opioid drugs use raises the question of the impact of these substances on psychocomportemental symptoms, leading to academic difficulties. Conversely, we may hypothesize that schooling difficulties due to ADHD could be at the root of benzodiazepine and opioid drug use. Indeed, it is known that the use of psychoactive substances, including benzodiazepines and opioid medications, can lead to attentional difficulties, which may have a positive influence on scores on the ASRS, thus supporting the first hypothesis. However, we also found that benzodiazepine and/or opioid medication use were associated with childhood ADHD symptoms, thus supporting the second hypothesis. As the WURS and ASRS are only screening scales and cannot be used to diagnose ADHD, those results underline the need to screen at-risk populations and then to perform further clinical evaluation.

In our results, a high proportion of drug users had psychiatric follow-up, which seems to highlight a significant level of distress in the student population. It remains unclear whether the combination of precarious mental health and difficulties at school can constitute a breeding ground for substance abuse. A longitudinal study in the USA found an independent association between mental health difficulties and academic performance in adolescents; hyperactivity disorders at this age were also described as predisposing to poorer academic performance^37^. Likewise, Weyandt et al. showed in 2016 that students with ADHD performed less well on executive functions than their peers, and all the more so if there was an associated psychiatric comorbidity^38^. Interestingly, benzodiazepine use was inversely associated with addiction follow-up, suggesting that users of these substances may be less inclined to start addiction treatment, perhaps due to a lack of psychoeducation on the effects of dependence.

We also found an association between paid student internships and the use of benzodiazepines alone or in combination with opioids. For these students, it was their only source of income, raising the question of financial stress. In 2019 in the UK, a literature review explored the issue of financial stress among students and the link with their mental health^39^. Financial difficulties were shown to be cross-sectionally associated with mental health disorders in general among students, however, the results remained mixed and the evidence limited on larger samples and in longitudinal studies^39^. It would be interesting in the future to explore this question in relation to substance use among students.

Our results concerning the use of associated substances are worrying in terms of the number of young people who have already experimented with poly-drug use since the start of their studies. In the literature, Weyandt et al. reported that the students most at risk of opioid misuse were those with concomitant use of alcohol and illicit substances^23^. Similarly, Maust et al. found that in almost all cases, past-year use of tobacco, alcohol, cannabis, heroin, opioid drugs or prescription stimulants was associated with benzodiazepine use^24^. Regular consumption of tobacco, alcohol or cannabis could be targeted as markers for identifying young people most at risk of prescription drug misuse in prevention programs.

The question of the combined use of benzodiazepines and opioid medications is important because in our results, combining users seem to be more concerned by psychiatric follow-up, ADHD symptomatology and grade repetition. Whereas previous data evaluated the combined use of benzodiazepines and opioid medication, only a few studies have specifically explored the subject in the student population. The risk factors associated with the use of benzodiazepines and opioid drugs identified in our study could enable us to develop appropriate prevention programs. First of all, it seems important to provide healthcare professionals with the best possible training in the early identification of young people at risk of addiction, in the screening and diagnosis of ADHD, and in weighing up the benefit-risk balance when prescribing drugs at risk of addiction. Secondly, the psychoeducation of young people and their supervisors, through targeted addictology prevention programs, seems highly relevant. Several mental health prevention programs for young people have proved their worth^6,40–42^.

We should acknowledge certain limitations of this study: (i) the monocentric design, (ii) the response rate that may have induced self-selection bias, and (iii) the self-reported questionnaires, which may be the source of recall or prevarication bias. The results may also have been influenced by the global SARS-CoV-2 pandemic, as the study was conducted at the time of the third lockdown in France. It will be interesting to see how consumption evolves outside the pandemic context.

In all, our results underline the need for better-targeted prevention programs in high schools and university campuses concerning medication misuse and ADHD screening in at-risk populations.

## Data Availability

The datasets used and/or analysed during the current study available from the corresponding author on reasonable request.
